# Marine Mammal Impacts in Exploited Ecosystems: Would Large Scale Culling Benefit Fisheries?

**DOI:** 10.1371/journal.pone.0043966

**Published:** 2012-09-06

**Authors:** Lyne Morissette, Villy Christensen, Daniel Pauly

**Affiliations:** 1 Institut des Sciences de la Mer de Rimouski, Université du Québec à Rimouski, Rimouski, Québec, Canada; 2 Fisheries Centre, University of British Columbia, Vancouver, British Columbia, Canada; National Oceanic and Atmospheric Administration/National Marine Fisheries Service/Southwest Fisheries Science Center, United States of America

## Abstract

Competition between marine mammals and fisheries for marine resources—whether real or perceived—has become a major issue for several countries and in international fora. We examined trophic interactions between marine mammals and fisheries based on a resource overlap index, using seven *Ecopath* models including marine mammal groups. On a global scale, most food consumed by marine mammals consisted of prey types that were not the main target of fisheries. For each ecosystem, the primary production required (PPR) to sustain marine mammals was less than half the PPR to sustain fisheries catches. We also developed an index representing the mean trophic level of marine mammal's consumption (TL_Q_) and compared it with the mean trophic level of fisheries' catches (TL_C_). Our results showed that overall TL_Q_ was lower than TL_C_ (2.88 *versus* 3.42). As fisheries increasingly exploit lower-trophic level species, the competition with marine mammals may become more important. We used mixed trophic impact analysis to evaluate indirect trophic effects of marine mammals, and in some cases found beneficial effects on some prey. Finally, we assessed the change in the trophic structure of an ecosystem after a simulated extirpation of marine mammal populations. We found that this lead to alterations in the structure of the ecosystems, and that there was no clear and direct relationship between marine mammals' predation and the potential catch by fisheries. Indeed, total biomass, with no marine mammals in the ecosystem, generally remained surprisingly similar, or even decreased for some species.

## Introduction

Interactions between marine mammals and fisheries have received growing attention during the last decades [1]–[4]. Many studies have examined how fisheries may impact marine mammal populations [5]–[7], but the degree to which marine mammals compete for food with fisheries is still poorly known [5], [8]–[9]. Nevertheless, competition for fish resources may be a primary source of current and future conflicts [10]–[14]. Even though many authors now document a growing concern about the widespread decline of many marine mammal populations [8], [15]–[19], there is a serious need to address their competition with fishery for the same food resources.

Understanding how, where, and when marine mammals and fisheries compete is not an easy task [20]–[21]. First of all, detailed information on predation rates and how these relate to fluctuations in fish availability or marine mammal population size is lacking [22]–[23]. Furthermore, it is usually very difficult to observe marine mammal feeding and/or interacting with fisheries [24]–[25]. Quantifying their diets with estimation models (scats, stomach contents, fatty acids, etc.) is also problematic as diets can vary substantially over time and space [26]–[29]. Finally, even on the fisheries side, exhaustive data on yield and precise estimates on catches, bycatch (especially the commercially less important species), or discards are relatively hard to obtain [30]–[31].

Ecosystem models can be used address the trophic role of marine mammals in ecosystems, and their potential competition with fisheries. *Ecopath with Ecosim* (*EwE*) models are generally constructed to address fisheries questions, and merely consider the commercially important species, but in some cases marine mammal are included, providing a better representation of the trophic interactions in the upper trophic levels of ecosystems [32]–[33]. They then become useful to address questions about the competition between marine mammals and fisheries.

The present analysis has the following objectives: 1) to calculate the target species (prey or catch) overlap between marine mammals and fisheries; 2) to examine the global trophic impacts of marine mammals and fisheries on the commercially important species of each ecosystem; and 3) to simulate the extirpation of marine mammal populations and analyze the resulting changes on the structure of the food web. Also, we examine the impact of whales versus pinnipeds in ecosystems, in order to investigate if whaling and seal culling would have the similar impacts on ecosystems.

## Materials and Methods

### Modeling approach and ecosystem representation

*EwE* is a widespread software package widely used for the analysis of exploited aquatic ecosystems ([34], freely available through http://www.ecopath.org). This modeling approach creates a simple static model to describe the average interactions of the populations within an ecosystem during a certain period. The model assumes mass-balance, i.e., that we account for all energy flows in a food web. Such an approach is different and much easier to implement than attempts to model multispecies interactions such as multispecies virtual population analysis (MSVPA; [35]) for which an enormous quantity of catch-at-age data and stomach contents analyses is required [36].

While there is spectrum of models ranging from single species assessments through so-called ‘minimum realistic ecosystem models’ to very complex end-to-end models, *EwE* models generally represent a less-data intensive approach that allows evaluation of ecosystem-level questions with focus on the higher trophic levels [37]. In *EwE* models, input values (mainly biomass, production, consumption, diet composition and harvest) often are available for several species or groups in the ecosystem, and it is an approach allowing the construction and rapid evaluation of balanced ecosystem models [38]. *EwE* has the advantage of including all/most of the important species groups in contrast to minimum realistic models (MRM), and allows evaluation of unexpected indirect interactions that an MRM could miss.

For this study we used a series of ecosystem models. Each *Ecopath* model was based on mass balance principles, assuming that production of a given prey group (i) was equal to the biomass lost to fishing or export, predation, and natural mortality other than predation (other mortality). This mass balance can be expressed as:
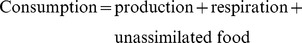
(1)and
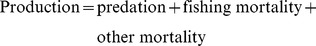
(2)where consumption is composed of consumption within the system and consumption of imports (i.e., consumption “outside the system”), and production may be consumed by predators, be exported from the system or contribute to the detritus. The terms of these equations may be replaced by:
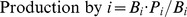
(3)

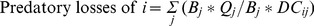
(4)and
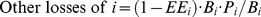
(5)


For any species or group of species of the system, this leads to the linear equation:
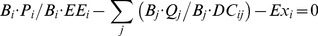
(6)where:

*i* indicates a component (stock, species, group of species) of the model;
*j* indicates any of the predators of *I*;
B*_i_* indicates the biomass of *I*;
P*_i_*/B*_i_* indicates the production/biomass ratio, which is equivalent to total mortality; (Z) under the most circumstances [39];
Q*_i_*/B*_i_* indicates the food consumption per unit biomass of *i*;
DC*_ij_* indicates the contribution of i to the diet of j (in terms of mass);
EE*_i_* indicates the ecotrophic efficiency of *i*, or the fraction of production that is consumed or caught within the system;
Ex*_i_* indicates the export of i from the system (by emigration or fisheries catches).

Algorithms in the model also allow for the estimation of one missing parameter (B*_i_*, Q*_i_*/B*_i_*, P*_i_*/B*_i_*, or EE*_i_*) in each group [38].

In *Ecopath*, several system indices (see below) are computed to describe the food web, its complexity, and the way trophic groups interact with one another. The software also allows dynamic simulations through the *Ecosim* module, a dynamic modelling approach for exploring past and future impacts of fishing and environmental disturbances [40].

*Ecosim* provides temporal simulations using the initial parameters of the *Ecopath* master equation (Eq. 7). It works with a couple of differential equations to estimate biomass fluxes as follows:
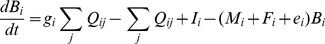
(7)where *dB_i_*/*dt* is the biomass growth rate of group (*i*) during the interval *dt*, *g_i_* is the net growth efficiency (production/consumption ratio), *I_i_* is the immigration rate, *M_i_* and *F_i_* are natural and fishing mortality rates of group (*i*), *e_i_* is emigration rate [41]. *Ecosim* describes the interactions between predators and prey by attributing a density-dependent term (‘vulnerability’) for each of these interactions. This vulnerability parameter sets the maximum increase in predation mortality a given predator can cause on a given prey [40].

Because there is good coverage based on the same modelling methodology available from throughout the world's oceans, we chose *EwE* models as our sample units to quantify and analyze the impact of marine mammals in marine food webs. This approach is important because it also represents a rational way of quantifying the trade-offs between sustainable exploitation of natural marine resources and conservation of charismatic fauna [42]. The models also have the advantageous possibility of being validated to conventional stock assessment data or surveyed biomass estimates [33].

**Figure 1:**
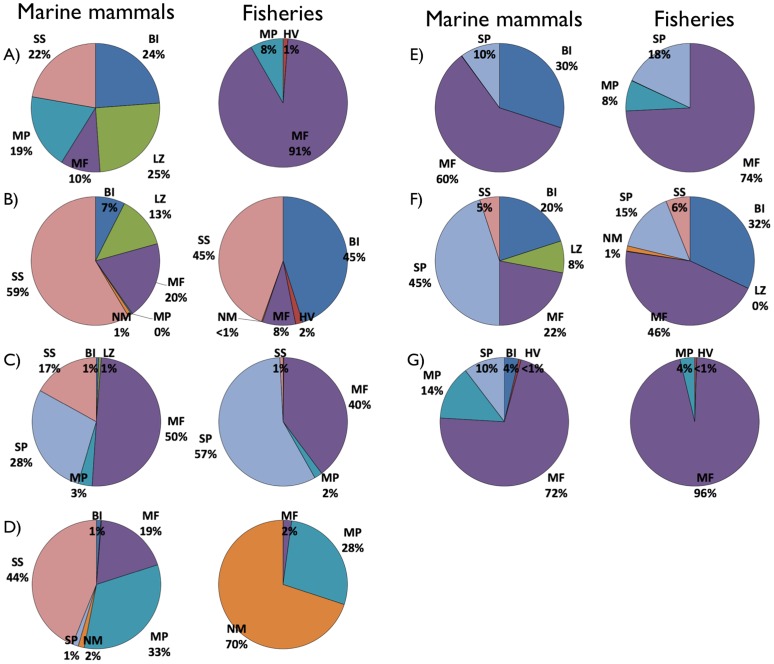
Location of the ecosystem modeled with *Ecopath* and used for this analysis.

Seven models are part of this analysis, selected in terms of their location and the quality of their documentation (Table 1). Particular effort was made to cover both northern and southern hemispheres, in an attempt to have a wide coverage of the world's oceans for the global extrapolation. Kaschner and Pauly [43] have shown that the prominent hotspots of overlap and potential competition between marine mammals and fisheries include the Bering Sea where the potential negative impacts of the US groundfish fisheries on the endangered western population of Steller sea lions (*Eumetopias jubatus*) have been of great concern [44]–[45], the Benguela system off southwest Africa with the potential impacts of the increasing population of South African fur seals on the hake stocks [46] and the east coast of North America where the largest annual marine mammal cull worldwide is in part being justified based on the perception that the growing harp seal (*Phoca groenlandica*) population impedes the recovery of the northwest Atlantic cod (*Gadus morhua*) stocks (see review in [47]). To these three ecosystems, we added other ecosystems models that didn't necessarily featured a marine mammal-fisheries issue, but that were characterized by important and well-documented fisheries and marine mammals: the eastern tropical Pacific Ocean; the North Sea; the Gulf of Thailand and the Strait of Georgia (Figure 1). The seven models show different levels of aggregation (how the species are grouped to represent the whole foodweb) and cover from 26 to 40 trophic groups. They were selected because they included marine mammals and used high-pedigree data to describe their diet of these groups. The ‘pedigree’ of an Ecopath input is here understood as a coded statement categorizing the origin a given input (i.e., the type of data on which it is based), and specifying the likely uncertainty associated with the input, for diet data, it varies between 0 (general knowledge) to 1 (qualitative, detailed, diet composition) for diet composition [48] (Table 1). They were also selected based on the fact that they have been shown to reproduce well the past patterns of change in relative biomass of major species given historical disturbance patterns (fishing mortality rates and/or effort over time, and in some cases changes in oceanographic or nutrient-loading indices of relative primary productivity). For these seven ecosystems, an *EwE* model was obtained from the scientists who created it, and verified for its ability to reproduce observed time series of biomass changes (Table 1).

**Table 1:** *Ecopath* models used for analyses of marine mammals consumption.

Ecosystems	Area covered (km^2^)	No of trophic groups	Marine mammals groups	Average pedigree or marine mammals' diets	Reference
Eastern Bering	484,500	26	1) Baleen whales	0.7	National Research
Sea			2) Toothed whales		Council [30]
			3) Sperm whales		
			4) Beaked whales		
			5) Walrus and bearded seals		
			6) Other seals		
			7) Steller sea lions		
Northern Gulf	103,812	32	1) Cetaceans	0.6	Morissette et al. 2003
of St.			2) Harp seals		[109]
Lawrence			3) Hooded seals		
			4) Grey seals		
			5) Harbour seals		
Benguela	179,000	32	1) Seals	0.7	Shannon et al. 2004 [78]
system			2) Cetaceans		
Eastern	32,800,000	39	1) Baleen whales	0.8	Olson and Watters [93]
tropical Pacific			2) Toothed whales		
			3) Spotted dolphins		
			4) Meso. dolphins		
North Sea	570,000	32	1) Seals	0.7	Christensen et al. 2002 [77]
Gulf of Thailand	101,384	40	1) Marine mammals	0.5	FAO/FISHCODE 2001 [110]
Strait of	6,900	27	1) Transient orcas	0.6	Martell et al. 2002 [111]
Georgia			2) Dolphins & Resident orcas		
			3) Seals		
			4) Sea lions		

### Ecosystem indicators

For each model, a comparison of the *Ecopath* outputs for food consumption by marine mammals *versus* the catch by fisheries was performed. The estimated annual catches (i.e., ‘food consumption’) of fisheries and marine mammals was calculated for each ecosystem. We also compared the estimated catch composition of the fisheries to the diet of marine mammals. Finally, the primary production required (PPR) to sustain fisheries was compared with the PPR to sustain marine mammals groups.

The mean trophic level of marine mammals' consumption (TL_Q_) and of fisheries catches (TL_C_) were derived from *Ecopath* outputs. TL_C_ is an indicator of the ecosystem health and the state of the fisheries [49], based on Lindeman's [50] concept of trophic levels, and calculated as:
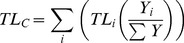
(8)where Y*_i_* is the total landings of species *i* (in tonnes), ΣY is the sum of landings for all species, and TL*_i_* is the trophic level for species *i*, which can be fractional as suggested by Odum and Heald [51].

Similarly, the trophic level of consumption (TL_Q_) by marine mammals was computed:
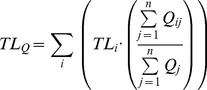
(9)where Q*_ij_* is the consumption of prey *i* (in tonnes) by marine mammal *j*, Q*_j_* is the total consumption of all species by marine mammal *j*, and TL*_i_* is the trophic level for species *i*. This equation represents the average trophic level on which marine mammals feed, i.e., the average TL of each species, multiplied by their proportion in the consumption matrix (tonnes·km^−2^·year^−1^ that marine mammals consume).

Using estimates of fisheries catches and marine mammal consumption, the assessment of overlap between marine mammal and fisheries for each ecosystem was performed using an equation derived from Kaschner and Pauly [43]:
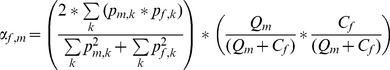
(10)where *α_f,m_* is the quantitative overlap between a fishery *f* and a marine mammal group *m* in the ecosystem, and the first term expresses the qualitative similarity in diet/catch composition between the marine mammal group *m* and fisheries *f* sharing the resource or food type *k*, with *p_m,k_* and *p_f,k_* representing the proportions of group *k* in the diet of marine mammals *m* or the catch by fishery *f*. This term is multiplied by the product of the proportion of total food consumption by marine mammals *Q_m_* and the proportion of total fisheries catches *C_f_* in the ecosystem. This index scales from 0 (no overlap) to 0.250 (identical resource). When resource use is identical between two groups, the first term of equation 10 is equal to 1, and each proportion of the second term is 0.5 (or 0.25 for the product). In order to make ecosystems comparable despite their different trophic structures, overlap by food types was also calculated, based on food categories that were first described in Pauly et al. [52]: benthic invertebrates (all crustaceans except krill, seasquirts, bivalves, gastropods, octopus, etc.), large zooplankton (mainly krill), small squid (mantle length <50 cm), large squid (mantle length > = 50 cm), miscellaneous fishes (Fishbase [53] habitat attributes: demersal, benthic, benthopelagic, bathydemersal, reef-associated, pelagic >80 cm), mesopelagic fishes, small pelagic fishes (fishbase attributes: pelagic <80 cm). The details on how the trophic groups of each model were classified into these different food categories are given in Table S1.

The mixed trophic impact (*TI*) analysis of *Ecopath* was used to compare the ‘with/without’ impact of predation by marine mammals on the whole ecosystem [54]. This quantifies all the direct and indirect trophic impacts of all groups in the system:
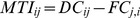
(11)where DC*_ij_* is the diet composition term expressing how much *j* contributes to the diet of *i*, and FC*_j,i_* is a host composition term giving the proportion of the predation on *j* that is due to *i* as a predator. When calculating the host compositions, the fishing fleets are included as ‘predators’.

Beneficial predation refers to a situation where a predator may have a direct negative impact on its prey, which is counterbalanced by indirect positive effects through the consumption of other predators and competitors of the prey [14]. It was calculated as the percentage of the overall trophic impact by marine mammals that is positive for any prey group of this predator.

### Dynamic simulations

In *Ecosim*, the predator-prey relationships are based on the foraging arena theory, dividing the prey biomass into vulnerable and invulnerable pools [55]. The vulnerability parameter (*v*) represents the transfer rate between these two pools, and has implications for how a given predator will impact predation mortality for a given prey, and can range from 1 to ∞. Low vulnerability factors (e.g. = close to 1) imply that an increase in predator biomass will not cause any noticeable increase in the predation mortality the predator will cause on the given prey. High vulnerability factors (e.g. = 100) contrarily indicate that if the predator biomass is for instance doubled, it will cause close to a doubling in the predation mortality rate on a given prey. This then relates directly to assumptions about the carrying capacity for the predator in question [40], and *Ecosim* predictions are very sensitive to this parameter [4]. The default vulnerability (2.0) assumes that each predator group can at most increase the predation mortality they impose on their prey by a factor of 2.0, while a lower value implies a donor-driven density-dependent interaction, and a much higher value involves a predator-driven density-independent interaction, in which predation mortality is proportional to the product of prey and predator abundance (i.e., Lotka-Volterra).

Here, we used a set of models whose vulnerabilities were derived by fitting to historical data following Walters et al [56]. In order to quantify the potential impact of marine mammal predation on the ecosystem, and to examine if there really is strong competition with the fisheries, *Ecosim* simulations were run for 22 to 89 years, depending on the time series available. The seven ecosystem models were first analyzed with *Ecosim* using time series of fishing mortality (F) to see which groups' biomass decline or increase over time. The models covered different sources of fishing mortality or fishing fleets (trawls, long lines, coastal, deep-water, whaling, etc), which were combined in every model to see the overall effect of fisheries on the entire ecosystem. A first simulation was done with the original ecosystem structure (and original catches of fish and marine mammals), while a second was performed with a very high catch of marine mammals, with the purpose of driving them extinct. Vasconcellos et al. [57] showed that for fish species, a 5-fold increase in fishing mortality leads to serious depletion. Also, such an extreme scenario is routinely applied to many fish populations and often associated with stock collapse [58]. Consequently, an F value of 1.0 year-1 (representing an average five-fold increase for marine mammal species that were already hunted) was applied to each marine mammal group in the models. The higher values of F were kept constant for the first 20% of the time series, and then returned to the baseline, with the model running for the remaining 80% of the time.

## Results

### Total food consumption by marine mammals *vs* fisheries catches

At the global scale, when considering all ecosystems at once, the major source of overlap between marine mammals and fisheries for all models combined was for ‘miscellaneous fish’ (i.e., demersal; benthic; benthopelagic; bathydemersal; reef-associated habitat & common length <150 cm; or pelagic habitat & common length >60 cm and <150 cm) and ‘small pelagic’ (pelagic habitat & common length <60 cm). Marine mammals' consumption was diversified and represented a great array of marine organisms (36% of miscellaneous fish, 21% of small squids, and approximately equal proportions [10–16%] of benthic invertebrates, large zooplankton, meso-pelagic, and small pelagic fish), while fisheries catches were concentrated at 51% on ‘miscellaneous fish’, 13% of small pelagic fish, and 11% of benthic invertebrates. While marine mammals could consume different prey groups (mainly large zooplankton, cephalopods, small pelagic fish and macrobenthos), fisheries in the seven studied ecosystems combined were mainly targeting small crustaceans such as shrimp, pelagic fish (redeye, *Etrumeus whiteheadi*; redfish, *Sebastes* spp.; anchovy, *Eugraulis capensis*; sprat, *Sprattus sprattus*), and demersal species such as hake species, lingcod (*Ophiodon elongates*), and sandeel (*Ammodytes tobianus*). The detail of each ecosystem is given below.

**Figure 2:**
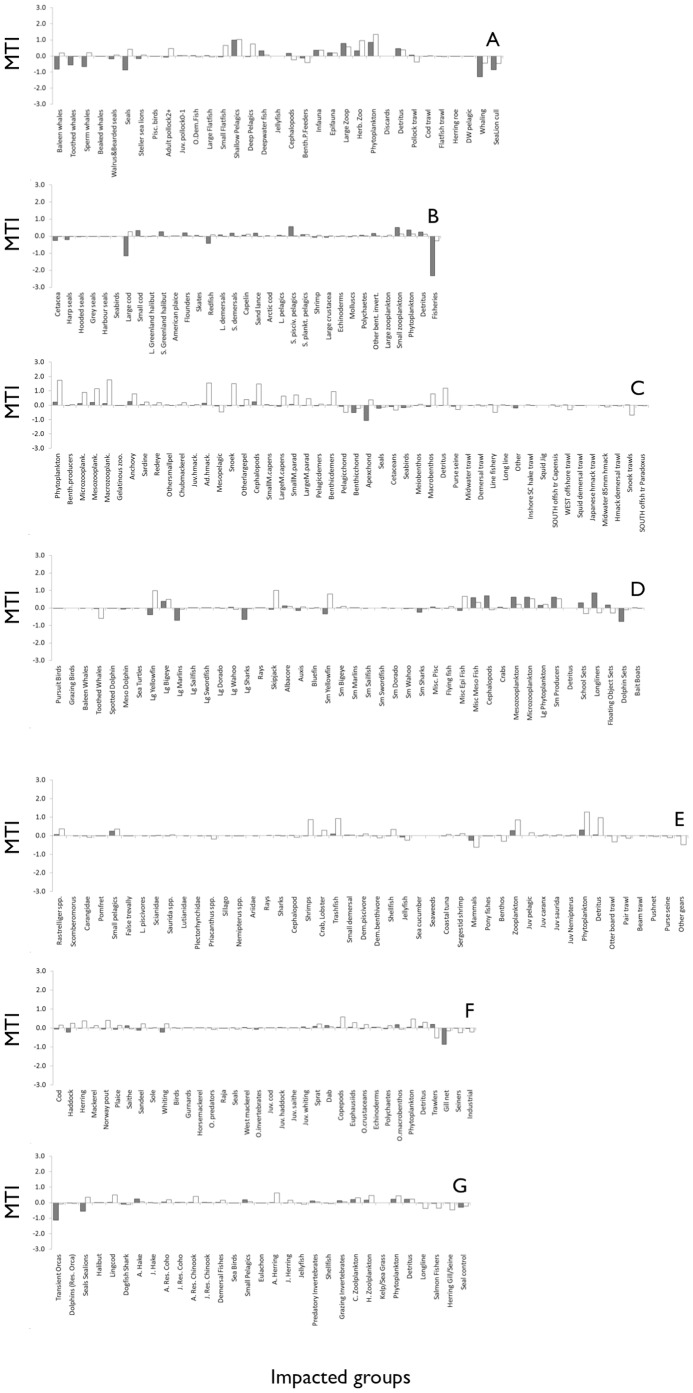
Estimated mean annual catch and food consumption by food types expressed as proportions of total amounts taken (t·km^−2^) in the Eastern Bering Sea (A), Gulf of St. Lawrence (B), Benguela (C), Eastern tropical Pacific (D), Gulf of Thailand (E), North Sea (F), and Strait of Georgia (G) ecosystems. Food types categories defined by Pauly et al. 1998*a*): Non-marine mammal food (NM), miscellaneous fishes (MF), small pelagic fishes (SP), benthic invertebrates (BI), small squids (SS), large squids (LS), mesopelagic fishes (MP) large zooplankton (LZ), higher vertebrates (HV).

In the Eastern Bering Sea system (Figure 2A), all fisheries catches fell into three types: mostly miscellaneous fishes (91%), but also mesopelagic fishes and higher vertebrates. In contrast, these food types accounted for less than a third of marine mammal consumption, which was more diverse and principally composed of large zooplankton (25%), benthic invertebrates (24%), and miscellaneous fish. In the Northern Gulf of St. Lawrence (Figure 2B), miscellaneous fish were the main target, accounting for 32% and 74% for marine mammal consumption and fisheries catches, respectively. However, the remaining marine mammal consumption was shared between three important groups (small pelagics, benthic invertebrates, and large zooplankton), while the fishery mainly caught miscellaneous fish (cod, redfish, and large Greenland halibut), benthic invertebrates (shrimp, crab, and molluscs) and small pelagics (herring). Marine mammal harvest (mainly seal hunt) also occurred in the Gulf of St. Lawrence, accounting for about 1% of the total catch. In the Benguela system (Figure 2C), more than 95% of all fisheries catches fell into three food types: small pelagic (57%), miscellaneous,(40%) and mesopelagic (2%) fishes. These food types were also the most important for marine mammals of this ecosystem (50% miscellaneous, 28% small pelagic, and 3% mesopelagic fish), whose diets also included an important proportion (17%) of small squids. In the eastern tropical Pacific model (Figure 2D) most fisheries catches were of two types: non-mammal food and mesopelagic, accounting for 70 and 28%, respectively. There, marine mammals fed on a variety of food types, mainly small squids (44%), mesopelagic (33%), and miscellaneous fish (19%). The North Sea model (Figure 2E) showed that about 75% of resources taken by marine mammals or by fisheries was composed of miscellaneous fish. However, the difference between marine mammals and fisheries was in what kind of miscellaneous fish they exploited. The main fishes eaten by marine mammals were dab and cod, while fisheries (at the time) mostly targeted Norway pout (*Trisopterus esmarkii*), sprat and sandeel. In the Gulf of Thailand (Figure 2F), marine mammals fed on a great variety of groups, while fisheries mainly caught miscellaneous fish (46%) and benthic invertebrates (32%). These two food types represented about a third of consumption by marine mammals, which was mainly composed of small pelagic fish (45%). ‘Trash fish’ (bycatch catches that are used in the production of fishmeal) was one of the most important miscellaneous fish to be taken by fisheries and marine mammals, but then the two competing groups differed as marine mammals consumed more small pelagic and benthos, and fisheries caught more shellfish and shrimp. In the Strait of Georgia ecosystem, almost all fish caught by the fisheries were miscellaneous fish (96%; Figure 2G), which also represented 72% of the consumption by marine mammal. The marine mammal prey was dominated by adult hake and demersal fishes, which was different from the miscellaneous fish (adult herring) caught by the fisheries.

Overall, the degree of overlap depended largely on the resolution of the marine mammals' prey and fisheries catches. Instances of direct overlap at the level of a trophic group (species of group of similar species) occurred in the Eastern Bering Sea (mainly for small flatfish, large flatfish, adult pollock [*Pollachius virens*], and other demersal fish), Gulf of St. Lawrence (planktivorous small pelagics, piscivorous small pelagics, shrimps and large crustaceans), Benguela (anchovy, redeye, and sardine [*Sardinops sagax*]), eastern tropical Pacific (small yellowfin tuna [*Thunnus albacares*], skipjack [*Katsuwonus pelamis*], and *Auxis* sp.), Gulf of Thailand (‘trashfish’, *Rastrelliger* spp., cephalopods, small demersals, and small pelagics), and Strait of Georgia (resident coho salmon [*Oncorhynchus kisutch*], resident chinook salmon, and lingcod). On the other hand, little overlap occurred between marine mammals prey and fisheries catches in the North Sea, even at the species level.

### Trophic interactions between marine mammals and fisheries

In most ecosystems, marine mammals fed on lower trophic level species than what was caught by fisheries (Table 2). Except for the northern Gulf of St. Lawrence and the Strait of Georgia, TL_Q_ had lower values than TL_C_. The largest discrepancy between these two values was observed in the eastern tropical Pacific, where TL_C_ is one trophic level higher than the TL_Q_ (4.70 *versus* 3.76).

**Table 2:** Mean trophic level of marine mammals' consumption (*TL_Q_*), Mean trophic level of fisheries' catches (*TL_C_*), primary production required (*PPR*), and overlap indices (α*_j,l_*) for marine mammals and fisheries, and connectance in our study areas.

Ecosystem model	*TL_Q_*	*TL_C_*	*PPR* Marine mammals' Q (% of total PP)	*PPR* Fisheries catch (% of total PP)	α_j,l_ per food type	α_j,l_ per trophic group	Connectance
Eastern Bering Sea	2.83	3.42	31.8	53.9	0.031	0.006	0.274
Gulf of St. Lawrence	3.24	3.71	9.8	18.4	0.161	0.034	0.298
Benguela	3.65	3.73	2.2	3.2	0.714	0.120	0.231
Eastern tropical Pacific	3.76	4.70	14.1	6.3	0.005	0.0003	0.218
North Sea	3.25	3.44	3.0	50.1	0.890	0.020	0.219
Gulf of Thailand	2.08	2.46	1.8	2.3	0.468	0.100	0.139
Strait of Georgia	3.36	3.25	6.0	6.7	0.163	0.024	0.250
**Global average**	**2.88**	**3.42**	**9.7**	**20.1**	**0.149**	**0.043**	**-**

The overlap index scales from 0 (no overlap) to 0.250 (identical resource). Connectance is an index of ecosystem complexity that represents the proportion of possible links between groups that are realized (links/species^2^).

The primary production required (PPR) to sustain marine mammal consumption was always lower than PPR to sustain the fisheries (Table 2). Globally, PPR for fisheries was twice as high as PPR for marine mammals' consumption (20% *vs.* 10%). Marine mammals of the Benguela system had the lowest PPR, requiring only 2.2% of total primary production of the system, compared to marine mammals in the Eastern Bering Sea, where the PPR was 31.8% of total primary production. For fisheries, the lowest value was in the Benguela system (3.2%), while the highest PPR for fisheries catch was in the Eastern Bering Sea (53.9%), closely followed by the North Sea (50.1%).

When marine mammals were considered all together, their resource overlap with the fisheries varied considerably within the seven ecosystems (Table 2). Ecosystems with higher resources overlap had a lower diversity of food groups caught/eaten by fisheries/marine mammals. Models with a very high proportion of miscellaneous fish had a higher resource overlap than systems where other food types were more important. When analyzed per trophic group instead than per food type, the overlap was always lower. Highest overlap value was seen in the Benguela system, while lowest overlap was in the eastern tropical Pacific. Interestingly, the North Sea ecosystem, which had the highest overlap per food type, had the third lowest value when overlap was calculated by trophic group. The number of trophic links in the ecosystem also had an effect on the resource overlap between marine mammal and fisheries. Indeed, food webs with lower connectance (less trophic links) generally showed higher overlap values (Table 2).

### Marine mammals, fisheries and their impact on the trophic structure

The mixed trophic impact evaluated direct and indirect trophic effects. In the Eastern Bering Sea both marine mammals and fishery had an overall negative impact on the entire ecosystem (MM = −2.98; fishery = −3.04). The groups that were mostly impacted by marine mammal consumption were deep-water fish, large flatfish and other demersal fish. Conversely, small flatfish, deep pelagics and flatfish trawl seemed to benefit from the presence of marine mammals while marine mammals and flatfish were the most impacted by fisheries (Table S1).

In the Northern Gulf of St. Lawrence, fisheries had an overall negative impact (−4.93) that was much higher than that of marine mammals (−2.93). The groups that were the most negatively impacted by marine mammals were large demersals, large pelagics, and Greenland halibut (*Reinhardtius hippoglossoides*; large and small). In contrast, skates, small demersals, shrimp and most benthic invertebrates seemed to benefit from marine mammals. All marine mammals and seabirds, large cod, shrimp, small demersals and most benthic invertebrates were negatively impacted by fisheries in the Gulf of St. Lawrence. Small Greenland halibut, large demersals, and small cod seemed to benefit from fisheries (Table S1).

In the Benguela model, fisheries' negative impact on the groups' biomass was larger than that of marine mammals by an order of magnitude (−0.105 vs −0.011, respectively). The groups that were most negatively impacted by marine mammal consumption were cape hake (*Merluccius capensis*), horse mackerel (*Trachurus capensis*), and cephalopods. Conversely, apex chondrichthyans, mesopelagics, and redeye seemed to benefit from marine mammals. The fleet “other fisheries” would also benefit from an increase in marine mammal biomass in terms of mixed trophic impacts (Table S1). The main groups negatively impacted by fisheries in the Benguela ecosystem were snoek (*Thyrsites atun*), sardine, other large pelagics, shallow-water cape hake, and deep-water cape hake (*Merluccius paradoxus*) (Table S1).

In the eastern tropical Ocean, the *TI* of marine mammals and fisheries showed that fishery (−1.869) and marine mammals (−2.334) had an overall negative impact on the whole ecosystem. Groups that were the most negatively impacted by marine mammal consumption were small bigeye (*Priacanthus arenatus*), small wahoo (*Acanthocybium solandri*), albacore (*Thunnus alalunga*), and skipjack. On the other hand, large bigeye, large dorado (*Coryphaena hippurus*), large wahoo, large sharks, rays, and flyingfish (*Cypselurus naresii*) all benefited from the presence of marine mammals, while fisheries negatively affected them. Some fisheries also benefit from the presence of marine mammals in the system: the horse mackerel fisheries (midwater and demersal fleets) and snoek trawls (Table S1) are examples. The *TI* also showed that groups that were the most positively impacted by the combined effect of all fisheries were mainly the juveniles of important commercial fish (wahoo, dorado, swordfish [*Xiphias gladius*], sailfish [*Istiophorus platypterus*], marlins, bigeye). When all the fisheries were grouped, their combined effect on the individual fishing fleets was mostly negative, suggesting a high level of competition between the fleets.

In the Gulf of Thailand, the *TI* of the fisheries was overall negative (−7.735), and much higher than that of marine mammals (−1.899). Marine mammals had larger negative impact on small pelagics, jacks (Carangidae), *Rastrelliger* spp., and on the purse seine fishery (Table S1).

In the North Sea model, the negative impact of fisheries (−7.79) was three times larger than the impact of seals (−2.22). Many of the groups that were seriously impacted by fisheries showed positive impact by seals; for instance, for gurnards (*Lepidotrigla* spp.), horse mackerel (*Trachurus trachurus*), sole (*Solea solea*), juvenile saithe (*Pollachius virens*), rays, and herring. Some groups were positively impacted by fisheries, such as dab (*Limanda limanda*), sandeel, juvenile haddock (*Melanogrammus aeglefinus*), juvenile cod, and birds. All of the groups (except for dab) were also positively impacted by seals. Negative impacts from seals were mainly observed for cod, saithe, plaice and dab (Table S1). Here again, the overall impact of all fisheries grouped together was damaging for each single fishing fleets.

In the Strait of Georgia, the *TI* analysis showed that while the overall trophic impact of marine mammals was near neutral (−0.83), there was a strong negative impact by fisheries on the food web (−7.79). This negative impact of fisheries affected almost all fish species in the ecosystem (Table S1), except for dogfish (*Squalus acanthias*), juvenile herring, and juvenile coho salmon, which seemed to benefit from fisheries. Marine mammals' negative impact on fish was always smaller than fisheries' impact on the same groups. Interestingly, the overall effect of marine mammal on fishing was positive for trawlers, gill nets, seiners and industrial fleets. Total marine mammals impact was also strongly negative for resident killer whales, seals and sea lions (Table S1).

### Dynamic simulations

**Figure 3:**
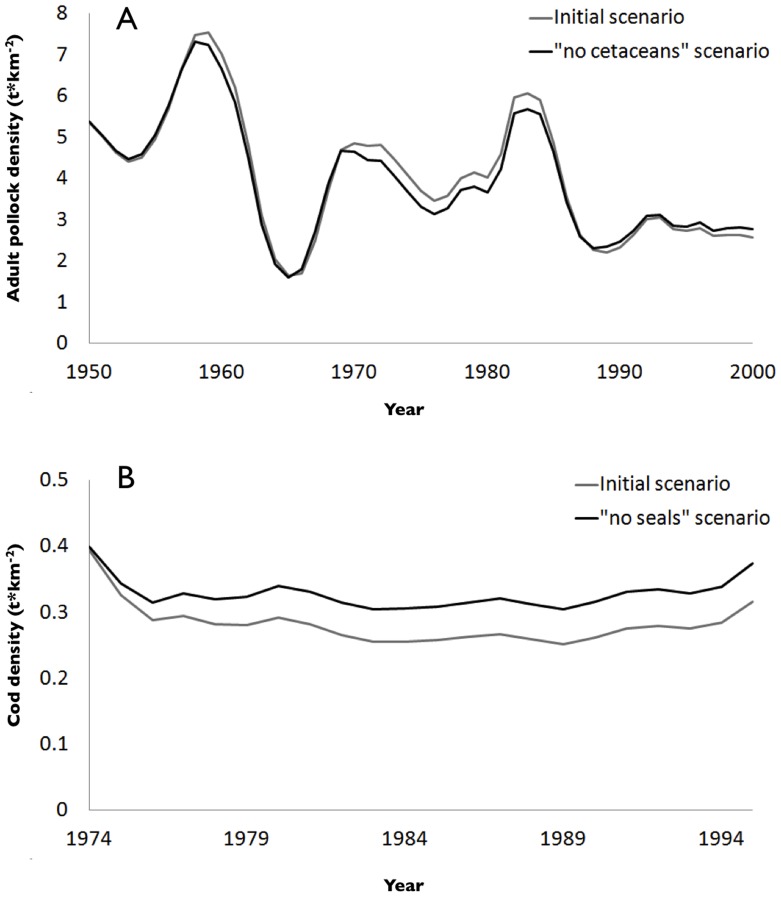
Change in commercially important fish biomass before (grey line) and after (black line) the simulated eradication of all marine mammals. Examples for pollock and cetaceans in the Eastern Bering Sea (A) and for cod and seals in the North Sea (B).

Different simulations were done for each model (eradication of all marine mammals, or of their component groups), and changes in biomass trends were recorded (see, e.g., Figures 3A and 3B). In both examples, the complete eradication of different groups of marine mammals created a change in the biomass trajectory of fish species, however this specific change was less than 15%, and could approach zero even after 50 years of simulations.

**Figure 4:**
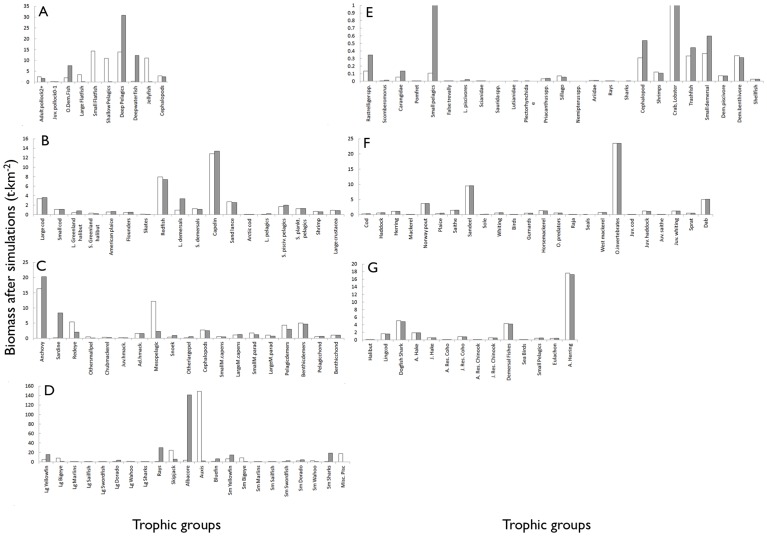
Biomass changes simulations with (white) and without (grey) marine mammals in the Eastern Bering Sea (A), Gulf of St. Lawrence (B), Benguela (C), Eastern tropical Pacific (D), Gulf of Thailand (E), North Sea (F), and Strait of Georgia (G) ecosystems.

After simulating marine mammal extirpation in the eastern Bering Sea ecosystem, there was an increase in biomass (compared to the simulation without marine mammal extirpation) in the other demersal fish, deep pelagics, deep-water fish, jellyfish, and cephalopods, but all other groups showed a decrease in biomass (Figure 4A). Over a period of 51 years, there was an overall decrease of 6% of total biomass if marine mammals were eradicated (B_tot_ = 316 t·km^−2^ with marine mammals, and 298 t·km^−2^ without them). This represents of course the complete extirpation of marine mammals biomass itself, but also seabirds, and other fish species such as small flatfish, as well as a critical biomass decrease for shallow pelagics (−99%), and epifauna (−97%). Our simulation also predicted that there were less fish to catch for the main species targeted by fisheries (adult pollock and shallow-water pelagics) without marine mammals (a decrease of 16% and 99%, respectively) (Figure 4A). Five fisheries out of eight suffered from a decrease in the biomass of their target species if there were no marine mammals in the ecosystem.

In the Gulf of St. Lawrence, when all seal and cetacean species were removed from the ecosystem, its structure changed. There was an explosion in the biomass of Greenland halibut, and an increase in large pelagic and demersal groups. Most groups that originally had lower biomasses appeared to increase their biomass after the extirpation of marine mammals. However, for commercially important groups, (e.g., adult cod, capelin, and small planktivorous pelagics), the increase was limited (Figure 4B). The most important fishery in terms of landings in the 1980s was for cod, redfish, small planktivorous pelagics (herring), and shrimp. Without marine mammals, most of these target species showed no significant change, or decreased slightly (Figure 4B).

On the one hand, in the Benguela system, the removal of the cetaceans and seals groups seemed to generate an explosion of sardine, as well as anchovy, snoek, other pelagics and seabirds. On the other hand, groups such as mesopelagics (−64%), other small pelagics (−27%), redeye (−20%), and pelagic demersals (−19%) showed decrease in biomass (Figure 4C). Increase in biomass was observed for snoek (47%), other large pelagics (28%), seabirds (27%), and large *M. capensis* (20%). The main targeted species in the Benguela ecosystem were anchovy, sardine, redeye (all caught by purse seine) and large deep-water Cape hake (caught by offshore trawl). When marine mammals were removed from the ecosystem, most of these commercially important fish ended up with less biomass than in the initial ecosystem, except for anchovy (Figure 4C). Out of 15 different fisheries in the Benguela ecosystem, eight underwent notable loss in the biomass of their target species after 25 years without marine mammals. Moreover, four of these were in the top-five fisheries with the most important catch at the beginning of the simulation.

In the eastern tropical Pacific, when marine mammals (here dolphins) were removed from the ecosystem, there was an increase in the biomass and hence predation of large fish such as wahoo (strongest increase in biomass; 248%), yellowfin tuna, bigeye, sharks, skipjack, albacore, bluefin tuna (*Thunnus orientalis*), and dorado (Figure 4D). The most important fish in terms of biomass (*Auxis* sp., flyingfish, and miscellaneous epipelagic fish) would decline if marine mammals were removed from the ecosystem (Figure 4D). However, commercially important species (especially bigeye, wahoo and skipjack), which tend to have lower biomasses, would benefit from the extirpation of marine mammals.

The Gulf of Thailand model showed that when all marine mammals were extirpated, this lead to an explosion in demersal benthivore species, but also to large variation in the biomass of small demersals, small pelagics and *Rastrelliger* spp. The remaining groups seemed to stabilize around equilibrium after the 24-year simulation. The most important groups in the Gulf of Thailand in terms of landings were ‘trash fish’, shellfish, shrimps, *Rastrelliger* spp., cephalopods, small pelagics, and crabs & lobsters. In a scenario without marine mammals in the ecosystem, most of these target species increased, except for shrimp, which was commercially very important (Figure 4E).

In the North Sea model, after the simulated seal extirpation (Figure 4F), the dynamics remained approximately the same. No fish species showed a clear increase in biomass. Their biomass may have been replaced by cod and saithe, which showed an increase in biomass following the decline of seals (Figure 4F). The main species targeted by the North Sea fisheries were adult Norway pout, sandeel, sprat, saithe, herring, whiting, and haddock. When marine mammals were absent from the system, *Ecosim* predicted that these fisheries caught about the same amount of fish than if marine mammals were present in the system (the largest changes were increases of 11% and 14% for whiting and saithe, respectively). Out of four fisheries, one (seiners, catching herring and mackerel) was definitely decreasing in terms of catch if there were no marine mammals in the ecosystem. In general, all trophic groups stayed at approximately the same level of biomass, with or without marine mammals in the ecosystem (Figure 4F).

Finally, in the Strait of Georgia, at the end of the simulation extirpating marine mammals, there was strong variations in halibut biomass, and the biomass of small pelagics, jellyfish, eulachon (*Thaleichthys pacificus*), and adult hake (*Urophycis tenuis*) came close to zero. All other groups appeared to stabilize at lower levels at the end of the 50-years simulation. The main targeted species were herring, resident Chinook salmon, lingcod, and resident Coho salmon. When marine mammals were removed from the ecosystem, most of these commercially important fish ended up with more biomass than in the initial ecosystem, except for herring, which decreased by 13% (Figure 4G).

### Differences between groups of marine mammals

In order to have noticeable effect on the biomass of commercially important fish, we would need to eradicate all marine mammals of the ecosystem (Table 3). However, even in that case, unexpected effects occurred, such as decreases of all commercially important fish in the Benguela and Strait of Georgia ecosystem. When removing only whale species, some ecosystems showed an increase in biomass for commercially important species, (e.g., Gulf of St. Lawrence), while some showed an (expected) increase in biomass, (e.g., predator fish and benthos in the Bering Sea). However, this effect was mainly done to the eradication of toothed whales, while baleen whales had almost no effect on these systems (Table 3). The eradication of marine mammals also had important impacts on fish that were not targeted by fisheries (Table 4). Indeed, most species that were not fished suffered from a decrease in biomass when marine mammals were removed from their respective ecosystem. This included depletion of forage fish in the eastern Bering Sea, and to a lesser extent a noticeable reduction of predatory fishes in the Gulf of St. Lawrence. Interestingly, the slight increases in biomass were more noted after eradicating baleen whales than any other marine mammal groups, while the most cases of a biomass decrease of non-exploited species was when eradicating pinnipeds and dolphins (Table 4).

**Table 3:** Percentages of change in biomass for unfished trophic groups of prey, by different marine mammals groups, in the seven study ecosystems.

Ecosystem model	Trophic groups	No MM	No pinnipeds	No dolphins	No whales	No toothed whales	No baleen whales
Bering Sea	Predator fish	-	-	-	-	-	-
	Forage fish	−97.0	−38.1	-	−54.9	−36.0	−10.0
	Cephalopods	−15.9	−18.3	-	16.9	0.7	17.2
	Benthos	−14.9	11.0	-	−9.4	−7.4	3.6
	Plankton	1.1	0.2	-	0.4	0.2	0.1
Northern Gulf of	Predator fish	−18.3	−18.8	-	−2.2	-	-
St. Lawrence	Forage fish	−5.1	2.4	-	−2.7	-	-
	Cephalopods	-	5.7	-	-	-	-
	Benthos	0.02	−0.6	-	0.1	-	-
	Plankton	−0.03	−0.3	-	−0.1	-	-
Benguela	Predator fish	4.9	9.8	-	2.4	-	-
system	Forage fish	-	-	-	-	-	-
	Cephalopods	-	-	-	-	-	-
	Benthos	−2.9	−2.3	-	−0.6	-	-
	Plankton	−6.5	−5.4	-	−3.1	-	-
Eastern tropical	Predator fish	−1.9	-	−1.6	-	−0.4	0.03
Pacific	Forage fish	−0.7	-	0.1	-	−0.9	0.1
	Cephalopods	5.1	-	5.1	-	−0.2	0.2
	Benthos	0.03	-	−0.02	-	−0.1	0.04
	Plankton	0.2	-	−0.01	-	0.1	0.02
North Sea	Predator fish	-	-	-	-	-	-
	Forage fish	-	-	-	-	-	-
	Cephalopods	-	-	-	-	-	-
	Benthos	−0.1	−0.1	-	-	-	-
	Plankton	−0.1	−0.1	-	-	-	-
Gulf of	Predator fish	-	-	-	-	-	-
Thailand	Forage fish	-	-	-	-	-	-
	Cephalopods	−2.1	-	-	-	-	-
	Benthos	0.2	-	-	-	-	-
	Plankton	0.1	-	-	-	-	-
Strait of Georgia	Predator fish	14.1	14.4	-	-	−3.7	-
	Forage fish	5.4	2.4	-	-	2.3	-
	Cephalopods	7.4	5.7	-	-	1.3	-
	Benthos	−0.7	−0.6	-	-	−0.1	-
	Plankton	−0.1	−0.3	-	-	0.1	-

**Table 4:** Percentages of change in biomass for commercially important trophic groups of prey, by different marine mammals groups, in the seven study ecosystems.

Ecosystem model	Trophic groups	No MM	No pinnipeds	No dolphins	No whales	No toothed whales	No baleen whales
Bering Sea	Predator fish	160.2	39.0	-	18.5	13.3	4.1
	Forage fish	−21.0	−33.6	-	−48.4	−45.6	0.5
	Cephalopods	-	-	-	-	-	-
	Benthos	53.1	15.4	-	38.0	35.4	−3.9
	Plankton	-	-	-	-	-	-
Gulf of St.	Predator fish	19.7	8.3	-	6.7	-	-
Lawrence	Forage fish	4.2	0.7	-	−23.67	-	-
	Cephalopods	-	-	-	-	-	-
	Benthos	0.01	−0.1	-	0.1	-	-
	Plankton	−9.0	−6.5	-	−3.9	-	-
Benguela	Predator fish	−8.9	−7.1	-	−5.1	-	-
	Forage fish	−4.5	−1.5	-	11.4	-	-
	Cephalopods	−8.7	−11.1	-	−15.4	-	-
	Benthos	-	-	-	-	-	-
	Plankton	-	-	-	-	-	-
Eastern tropical	Predator fish	8.9	-	1.5	-	7.1	0.2
Pacific	Forage fish	-	-	-	-	-	-
	Cephalopods	-	-	-	-	-	-
	Benthos	-	-	-	-	-	-
	Plankton	-	-	-	-	-	-
North Sea	Predator fish	2.0	2.0	-	-	-	-
	Forage fish	1.5	1.5	-	-	-	-
	Cephalopods	-	-	-	-	-	-
	Benthos	-	-	-	-	-	-
	Plankton	-	-	-	-	-	-
Gulf of	Predator fish	90.1	-	-	-	-	-
Thailand	Forage fish	181.2	-	-	-	-	-
	Cephalopods	74.1	-	-	-	-	-
	Benthos	3.1	-	-	-	-	-
	Plankton	−18.6	-	-	-	-	-
Strait of Georgia	Predator fish	−7.5	−6.0	-	-	−2.2	-
	Forage fish	-	-	-	-	-	-
	Cephalopods	-	-	-	-	-	-
	Benthos	-	-	-	-	-	-
	Plankton	-	-	-	-	-	-

**Figure 5:**
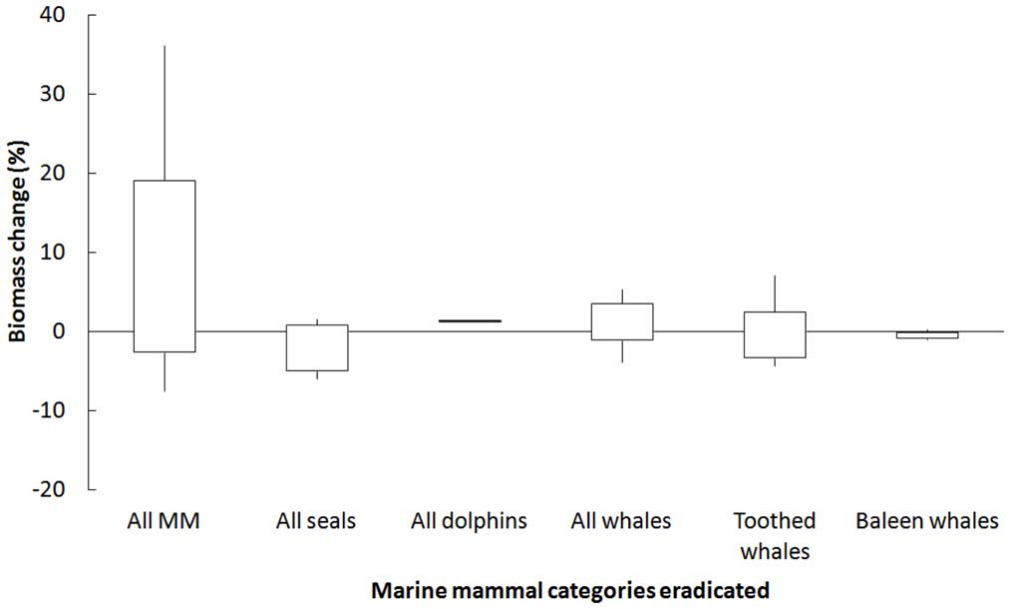
Total percentage of increase in the biomass of commercially important fishes when removing different groups of marine mammals. Boxes represent the lower 25% quartile up to the higher 75% quartile, while lines represent the smallest and the largest observations.

When all the simulations were pooled and analyzed by type of marine mammals (seals, dolphins, toothed whales, or baleen whales), the percentage of change in the biomass of commercially important fish (the groups with fishing mortality in the original models) increased differently according to the type of eradication simulated (Figure 5). When eradicating all marine mammals, the average response from all studied ecosystems showed the most important increase (Figure 5). However, the general response could vary from a slight decrease in biomass to an increase of about 20% of commercially important fishes. Similarly, when eradicating all whale species from the ecosystems, the overall response was a slight increase (less than 10%) in the biomass of commercially important fish. However, this was mainly driven by the removal of toothed whales, for which eradication leads to more chances of a positive effect (increase) on commercial fish species. When removing baleen whales from these systems, the response was almost null, but represented a negative effect on commercial fish biomass. Interestingly, the same effect was seen for seals, whose eradication would suggest an overall decrease of commercial fish biomass in most of the studied systems (Figure 5).

### Mixed trophic impact *versus* catch or consumption

**Figure 6:**
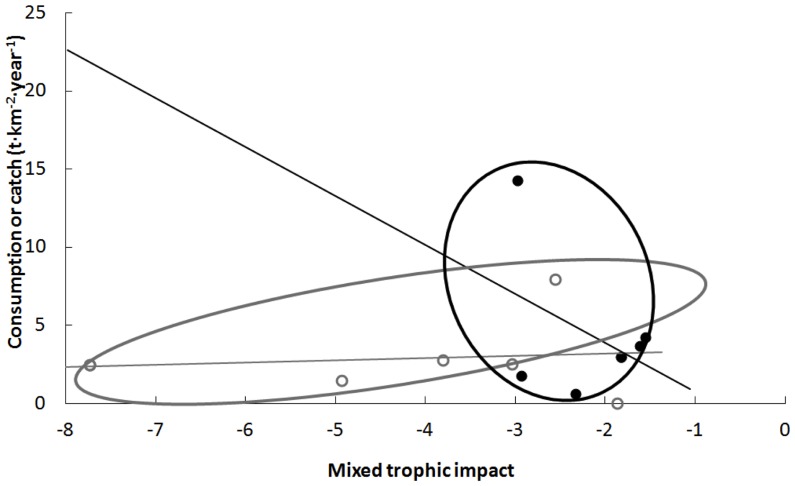
Total consumption by marine mammals (black dots) or total catch by fisheries (open dots) versus their respective overall mixed trophic impact for each studied ecosystem. Density ellipses represent the 90% confidence intervals.

When mixed trophic impacts were plotted against consumption (for marine mammals) or catch (for fisheries), it appeared that marine mammals consumed generally less than fisheries catch, and that their *TI* was less negative than that of fisheries for the same consumption or catch level (Figure 6). Moreover, the overall mixed trophic impacts of the marine mammals on the whole ecosystem became less negative with increasing consumption. This was a rather surprising result, to be discussed further below.

## Discussion

The trophic impact of marine mammals on food webs is generally seen as a direct relationship between the predators and their prey. Many studies [59]–[62] have addressed the question of the trophic role of top predators without taking into account the indirect trophic effects that they can have on their prey. The results presented here suggest that marine mammals can have important indirect effects on trophic structure. Therefore, our analysis offers a new perspective on the function of these predators in marine food webs, and their interaction with fisheries. This simulation modeling exercise proved to be a very informative aspect of our study, because it allowed us to capture the dynamic behavior of the system and the way in which trophic interactions might reconfigure when marine mammals are removed from these ecosystems.

We clearly see from results presented above that a change in marine mammal biomass can lead to important alterations in the structure of the ecosystem. In a time where marine ecosystems are overexploited [63]–[64], polluted [65]–[67] and subject to climate change [68], improving our ability to understand ecological processes involving marine mammals and fisheries becomes crucial.

### Strengths and weaknesses of the modeling approach

Studying marine mammals – fisheries interactions through a modelling approach can enhance our understanding of effect of relationships that would otherwise be very difficult to study. Our approach allowed to compare different ecosystems constructed using a common *EwE* framework. The level of detail included in the *Ecopath* model structure and equations is a real asset and represent a rigorous analytical framework [69].

There is a general, background controversy about appropriate levels of model complexity for the management of marine resources in the context of marine mammals – fisheries interactions [10], [69]–[70]. While single-species approaches to modeling fish stocks are more or less bound to conclude that marine mammals are detrimental to the stocks, because such models cannot incorporate the effects of indirect interactions, more complex multi-species models (which potentially can capture the behavior of the whole system) are more difficult to parameterize; also, *EwE* depends greatly on assumptions and parameter estimateswhich often cannot be rigorously tested by comparing with data.

Our results and conclusions are the product of the quality of data sources, assumptions and results of the seven *EwE* models that were used for this study. The level of aggregation, for example, was different from one model to another, depending on the original authors' choices, and the context or ecological question each model was built for. These differences in aggregation could lead to different prediction in dynamic models [71], and could be problematic for global comparisons. We did not re-aggregate the models but kept them with the same structure they had in their published version, mainly focusing on the fact that they covered marine mammal groups. This was therefore important to select the models that had the highest quality of data, and that showed a high accuracy in projecting biomass trends over time (i.e. they were fitted to time series in *Ecosim*).

Another potential issue with the way *EwE* models generally are constructed is that there is no possibility to discriminate between trophic levels that represent juveniles of larger fish or species that are truly occupying this trophic level at adult stage. Although this can be partly addressed with multi-stanza categorizations in *Ecosim* [34], most of the groups do not feature ontogenetic differences in the dynamics and diet. However, most of the marine mammals' diets used here are detailed to the species level, not often to the life stage or size of each prey. When diet information is entered in a non multi-stanza *Ecopath* model, a prey species, even juvenile, will be entered in the diet composition at the trophic level where the whole group (i.e., adult species) is. The groupings in *Ecopath* are done like this because information is based on adult diet. The juveniles are thus assumed to have the same diets than the adults, and their trophic levels represent the ‘potential trophic level’ they can reach at adult stage.

Some authors have warned modellers that caution must be taken in applying *EwE* models to marine mammal populations, because their life history is very different from most fish [69]. However here, the simulations we performed consisted in removing all marine mammals, and thus releasing their predation on other trophic groups in the ecosystem. The temporal variation of marine mammals' abundance itself was not addressed here (the simulated scenarios always presented how the system would react to the extirpation of marine mammal species, not with the possible causes and modalities of such extirpation). In *Ecosim* runs with original fishing effort (where marine mammals are present in ecosystems), we assume the vulnerabilities and the ecological reliability of each model is representative of the species dynamics and life cycles. The marine mammals groups present in the models are assumed to have realistic ecological parameters. However, the analysis done with these seven *Ecopath* models did not include any environmental effects that may affect ecosystem dynamics. We thus assumed that everything was explained by fisheries impact and species interactions, which is definitely not the case [72]. There is indeed strong evidence that climate change, for example, has an important impact on the availability of marine mammals' prey [73], as well as on their distribution and abundance [74]–[75].

One shortcoming of our approach is that we grouped all marine mammals together instead of analyzing pinnipeds, toothed whales, and baleen whales separately. Doing so, we masked the major differences in their feeding ecology. Also, analyzing marine mammals as a whole group could not show what effects their relative abundance have on the trophic impacts. The relative proportions of different marine mammal species would also affect the trophic level. For example, a large biomass of baleen whales, which would mainly consume krill, would significantly lower our estimate of trophic level of consumption. However, since we used the structure of the models directly as they were constructed, we could not separate these effects.

The calculated overlap index has the disadvantage of representing very large categories of species. Aggregation into large functional groups does not well represent the dynamics of the ecosystem. Thus, while the overlap index represents a good way to obtain a global and simple representation of the interaction between fisheries and marine mammals, the in-depth analysis of the structure of ecosystems remains crucial. The overlap index calculated at the species level showed that in the case of the North Sea, this can lead to opposite conclusions. Finally, the fact that the overlap index was calculated for marine mammals as a combined group presents, once again, a difficulty. Indeed, as most overlap with fisheries occurs among pinnipeds and dolphins, our estimates of the global overlap between fisheries and marine mammals may be underestimated.

The simulations are exploratory and represent extreme scenarios (the complete eradication of marine mammals). We did not explore any alternative or intermediate scenario. Consequently these last results should not be perceived as management strategies in any way.

### Resource overlap and trophic levels

On a global scale, most food consumed by marine mammals consists of prey types that are not the main target of fisheries, and whales seem to consume most of their food in areas where commercial vessels do not fish [43]. In areas where competition between marine mammals and fisheries is evident (identified as hotspots of resource overlap by Kaschner and Pauly [43]), our results show that the resource overlap is indeed higher than the global average presented in Kaschner and Pauly [43]. However, most overlap appears to occur between fisheries and larger, deep-diving toothed whales [43], so when marine mammals are analyzed overall, their overlap is not as strong as may be expected.

Depending on the ecosystem, the overlap between marine mammals and fisheries index involves different food types. In the North Sea, the Benguela, and the Strait of Georgia systems, marine mammals and fisheries compete mainly for the ‘miscellaneous fish’. This group includes demersal, benthic, benthopelagic and bathydemersal fish that are less than 150 cm, and pelagic fish that are between 60 cm and 150 cm [76]. For the purpose of this analysis, this prey group is clearly over-aggregated; and it is necessary to look at the composition of the ‘miscellaneous fish’ group in different ecosystems. In the North Sea, the Benguela and the Strait of Georgia ecosystems, there were important fisheries for larger fish [77]–[79], and the marine mammal species in these ecosystems are higher trophic-level predators, who mostly feed on these large fish [77]–[78], [80]. These ecosystems are quite different in terms of structure from the Gulf of St. Lawrence, where the intense fishing activity in the 1980s has lead to the depletion of most groundfish stocks, leaving mainly smaller planktivorous fish and crustaceans for fisheries and marine mammals [32], [81]. Consequently, in this ecosystem, small pelagics are the main overlapping resource. In the Gulf of Thailand, benthic invertebrates and miscellaneous fish are the food types that are most overlapping. Here again, the major development of trawl fisheries in the 1960s has lead to a shift to lower trophic levels such as ‘trash fish’ and shrimps [82], and most marine mammals are dolphins and whales that mainly eat smaller fish [83]. There may have been a time when fisheries and marine mammals did not overlap much in terms of food resources, but now that fisheries have moved down the food web [63], the target food types might have become very similar. Finally, in the eastern tropical Pacific Ocean, where the resource overlap is the lowest, there is not much competition between fisheries and marine mammal. Fisheries target mainly miscellaneous fish and large species such as tuna, and marine mammals feed mostly on small squids, mesopelagics and small pelagic fish. The number of trophic links in the ecosystem also has an effect on the resource overlap between marine mammal and fisheries. Indeed, food webs with lower connectance (less trophic links) tend to have higher overlap values.

Some ecosystems show major overlap between marine mammals and fisheries when analyzed by food types: the North Sea, Benguela and Gulf of Thailand most clearly. When the same analysis is done per trophic group (using the complete structure of the catch and the consumption matrix of marine mammals), the overlap index is reduced, but most ecosystems showing major overlap between marine mammals and fisheries remain the same. However, in the case of the North Sea, the overlap index calculated per trophic group is very low compared to the index calculated by food type, suggesting that there could be no overlap at the species level. However, diet studies on grey seals [84] show that sandeels, gadoids and other flatfish (targeted by fisheries) are important part of the diet. Grellier and Hammond [85] also showed that applying some digestion correction factors on grey seals diet in the North Sea reveal that some of its prey might in fact be similar to what is taken by the fisheries. This reduced overlap when we look at trophic group caught rather than food types might indicate that what seems to be similar in terms of type of fish caught by fisheries and eaten by marine mammals might in fact, when looked at it more specifically, be different species of fish. While *EwE* is suited to indicate general ecosystem properties, it is important to look at the species-specific results to have a clearer portrait of the situation.

Even at the trophic group level, the way the aggregations were done by the different authors who constructed the models seem to be important for our conclusions. Indeed, when fish species are aggregated onto trophic groups (i.e., large demersal feeders in the Gulf of St. Lawrence), chances are that even if this covers a group of species that are eaten by marine mammals and caught by fisheries, these could represent different fish species. Our study cannot address that level of detail, but this is definitely worth investigating. The real question in fisheries management is “are marine mammals stealing our fish?”. To this, our study provides part of an answer, showing that marine mammals and fisheries do not have the same targets. And the closer we look at aggregation levels, the clearer it gets that they target different species of fish.

Overall, landings from global fisheries are shifting gradually from large piscivorous fish toward smaller invertebrates and planktivorous fish [63]. Our results further show that overall the trophic level of marine mammals prey is lower than the trophic level of the catch (2.88 *versus* 3.42). Thus, as fisheries continue to move further down in terms of the trophic level of species caught, the competition for food resources with marine mammals may become more important. In ecosystems such as the eastern tropical Pacific where the mean trophic level of the catch still is high, the overlap with marine mammals is negligible. Interestingly, in the Strait of Georgia the marine mammals' consume prey at a higher trophic level than is caught by the fisheries. This is because of transient killer whales, which mainly feed on pinnipeds, at a very high trophic level.

Lately, some studies have stressed that whales globally consume 3 to 5 times more marine fish and invertebrates annually than is fished for direct human consumption or for reduction into fish meal and oil [86]. This situation, it is alleged, is not “in balance” with the world's increasing need for a stable food supply. Such arguments are used extensively to justify whaling activity, as it is shown in this quote by Mr. Masayuki Komatsu, formerly the executive director of the Japanese Marine Fisheries Research and Development Department, to BBC on “the forces that drive Japanese whaling”, 15 June 2006: *“Whale [are] abundant. The number of fish is falling while the number of whales is rising. Surely, the rapid increase in the whale population influences the level of fish stocks? We need to know more about it”*.

Our results show that there is no clear and direct relationship between marine mammals' predation and the potential fish catch in the world's oceans. Many whales do eat fish, but the species that they eat are not necessarily targeted by fisheries. In fact the global overlap of food resources, representing the main ‘hotspots’ of competition between marine mammals and fisheries [43], is relatively low. Moreover, as the simulation results showed, it is not that clear whether the extirpation of marine mammals in ecosystems would even increase the biomass of the fish targeted by most fisheries.

### Primary production required to sustain marine mammals and fisheries

In the ecosystems studied, the primary production required to sustain marine mammals represents an average of 9.7% of total primary production, less than half the PPR to sustain fisheries catch (20.1%). The latter value represents nearly three times the estimate by Pauly and Christensen [87] for global fisheries, as our analysis focuses on zones where fishing activity is intense.

In most ecosystems, PPR for marine mammals is lower than PPR for the catch, excepted in the eastern tropical Pacific Ocean. This model represents a very large area, and information about marine mammals (biomass, consumption rates, diet, production, etc.) is applied directly on the populations known to be within this area, while fisheries' effects might be more ‘diluted’ and less important in high sea.

The highest PPR for fisheries catch occurs in the North Sea (50%) and in the Eastern Bering Sea, (54%). PPR required by marine mammals is also the highest in the Bering Sea, followed by the eastern tropical Pacific. At the opposite end of the spectrum, the Benguela ecosystem, with the lowest PPR values for marine mammals as well as fisheries, is known to be a very productive ecosystem, due to the seasonal, wind-driven upwelling, and hence PPR is low [88].

### Comparing the mixed trophic impact of different marine mammals types (seals, toothed whales, small cetaceans, baleen whales)

While there is a growing concern about the potential impact of marine mammal populations on fisheries catches [11], [15], [43], [86], [89], our results show that the highest overlap occurs between fisheries and pinnipeds or dolphins. Whales, especially baleen whales, have the lowest impact on the ecosystem, and reducing their population would not benefit fisheries in any way. Even if it is known that baleen whales do eat fish in some areas [90]–[92], their impact on the ecosystem is still minimal, and they don't seem to be a threat to fisheries, even in high overlap areas.

### Comparing the mixed trophic impact of marine mammals and fisheries

The effect of marine mammals on their prey and consequently on available resources for fisheries is not only a direct predator-prey relationship. Rather their effect is also indirect, for example through feeding both on a prey and the competitors of the prey.

Even if negative for all studied ecosystems, the overall trophic impact of marine mammals on the different trophic groups of the ecosystem was always less strong than that of fisheries, except in the eastern tropical Pacific Ocean where fisheries target mainly large tunas (important predators of many trophic groups in the ecosystem), while marine mammals feed on a larger array of smaller prey [93].

For marine mammals, there is a paradoxical trend suggesting that the more they consume, the less they tend to reduce overall biomass. This is possible due to the fact that if marine mammals have to increase their consumption, they will feed on a wider array of prey and induce beneficial predation. In contrast, the mixed trophic impact of fisheries on the entire ecosystem is always strongly negative.

### What if there were no marine mammals?

The mixed trophic impact routine of *EwE* provides a first overview of the negative or positive impacts that marine mammals have in the systems when they are at equilibrium, and considering indirect effects. This represents a first step in understanding their trophic role. The second step is to explore if these effects are creating any reconfiguration in terms of structure in the long term, if marine mammals are removed. When the extirpation of marine mammals was simulated, the biomass of other species in the food web also changed. In some ecosystems, commercially important species increased significantly after the eradication of marine mammals, (e.g., halibut and large pelagics in the Gulf of St. Lawrence, tuna species in the eastern tropical Pacific, tuna and pelagic species in the Gulf of Thailand, and cod and plaice in the North Sea). However, when all commercial species were considered, there was no obvious benefit for the fisheries. Indeed, total biomass, with no marine mammals in the ecosystem, remained generally and surprisingly similar or even decreased (as it is the case with the Eastern Bering Sea and the Gulf of St. Lawrence). Indeed, the extirpation of marine mammals may lead to reduced abundances of commercial important fish in some ecosystems. Cape hake, sardine, redeye in the Benguela upwelling, and herring in the North Sea and in the Strait of Georgia decreased when marine mammals were removed from these systems. In the case of the Gulf of Thailand, the Plectorhynchidae group became totally depleted when marine mammals were absent. On the other hand, when species or groups increased as a result of the extirpation of marine mammals in the ecosystem, these species or groups were not necessarily the most important commercially (deep-water fish, jellyfish and cephalopods in the Eastern Bering Sea; cephalopods, juvenile pelagics, or juvenile carangids (jacks) in the Gulf of Thailand). Finally, when commercially important species increased following the extirpation of marine mammals, it was not necessarily a stable equilibrium [94]. There might be more fish to catch, but once overfished, these ecosystems could become unstable and at risk of severe losses in biodiversity.

In certain areas, (e.g., in Eastern Canada), there has been a heated debate on culling marine mammals in an attempt to rebuild stocks of once commercially important fish species, notably of cod [95]–[96]. In that particular case, at least for the Gulf of St. Lawrence ecosystem, our results suggested that culling of marine mammals would not have led to recovery of the stocks of cod, nor otherwise benefited the commercial fishery. This corroborates the findings of Trzcinski et al. [97], who suggested that even the complete removal of grey seal predation in the eastern Scotian Shelf (Northwest Atlantic) would not assure the recovery of the cod population, given the high levels of other sources of natural mortality.

### Are marine mammals a threat to fisheries?

Even in areas where there could be a competition between marine mammals and fisheries, the problem is mostly due to human use of marine resources. Over time, we have exploited and depleted the best marine resources, and now we are turning to what is left. In the process, we have moved from a zone of ‘no conflict’ to an area of higher overlap, as result of changing fisheries, and the collapse of overexploited large predatory fish.

At the International Whaling Commission, it has been proposed that baleen whales were a threat to fisheries, and that they should be culled. Regrettably, in this debate, it is difficult to assess whether it is based on any scientific evidence, considering the lack of evidence for existing large-scale competition between whales and fisheries [43], the well documented fact that the world's oceans increasingly are overexploited [63], [98]–[102], and the unpredictable consequences of culling [103]–[105]. Moreover, the areas where this argument is used the most are the Caribbean and Northwest Africa (L. Morissette, unpublished data), two regions where baleen whales are breeding, and where they reduce their consumption rate to about 10% of what it is in their high latitude feeding areas [106].

## Conclusions

Our analysis identified that marine mammals are important top predators in marine ecosystems, and that they play an important role in structuring the trophic relationships within food webs. Our results showed that even in hotspots of competition between marine mammals and fisheries, the overlap for food resources was lower than earlier presumed. Our results confirmed the findings of Kaschner and Pauly [43], who suggested that even the complete eradication of all marine mammals, from all oceans, would likely not increase fisheries catches. Hence, large-scale culling, as advocated in various Japanese studies (see, e.g., [86]) would probably not increase fisheries catches.

This study has focused on the top-down influences of marine mammals and fisheries on the fish species in different ecosystems. Although ‘bottom-up’ changes were not investigated here, their effects might just be additive and alter even more the structure of ecosystems. This is particularly true in the actual context of climate change, which can affect the productivity of the world's oceans [107]–[108]. There is still much debate about this idea and it will be important to find different ways of addressing this issue. The analysis presented here provided an insight into the problem, but further work on this needs to be pursued.

## Supporting Information

Table S1 Trophic groups of the seven ecosystem models used in this study and how they fall into food types categories defined by Pauly et al. [75]: Non-marine mammal food (NM), miscellaneous fishes (MF), small pelagic fishes (SP), benthic invertebrates (BI), small squids (SS), large squids (LS), mesopelagic fishes (MP) large zooplankton (LZ), higher vertebrates (HV). The mixed trophic impact (MTI) of marine mammals and fisheries is given for each impacted trophic group.
(DOCX)
