# Supplementary material for: Marine Mammal Impacts in Exploited Ecosystems: Would Large Scale Culling Benefit Fisheries?
Source: PLoS One. 2012 Sep 6;7(9):e43966. doi: 10.1371/journal.pone.0043966 (PMC3435392; doi:10.1371/journal.pone.0043966)
Supplement: Table S1 — Trophic groups of the seven ecosystem models used in this study and how they fall into food types categories defined by Pauly et al. [75]: Non-marine mammal food (NM), miscellaneous fishes (MF), small pelagic fishes (SP), benthic invertebrates (BI), small squids (SS), large squids (LS), mesopelagic fishes (MP) large zooplankton (LZ), higher vertebrates (HV). The mixed trophic impact (MTI) of marine mammals and fisheries is given for each impacted trophic group. (DOCX) [file pone.0043966.s001.docx]

Table S1. Trophic groups of the seven ecosystem models used in this study and how they fall into food types categories defined by Pauly et al. 1998*a*): Non-marine mammal food (NM), miscellaneous fishes (MF), small pelagic fishes (SP), benthic invertebrates (BI), small squids (SS), large squids (LS), mesopelagic fishes (MP) large zooplankton (LZ), higher vertebrates (HV). The mixed trophic impact (MTI) of marine mammals and fisheries is given for each impacted trophic group.

| **Ecosystem model** | **Trophic group** | | **Food type** | **MTI by marine mammals** | **MTI by fisheries** |
| --- | --- | --- | --- | --- | --- |
| **Eastern Bering Sea** | 1 | Baleen whales | HV | -0.494 | -0.500 |
|  | 2 | Toothed whales | HV | -0.524 | 0.017 |
|  | 3 | Sperm whales | HV | -0.575 | -0.433 |
|  | 4 | Beaked whales | HV | -0.176 | 0.163 |
|  | 5 | Walrus & Bearded seals | HV | -0.490 | -0.494 |
|  | 6 | Seals | HV | -0.515 | -0.482 |
|  | 7 | Steller sea lions | HV | -0.505 | -0.460 |
|  | 8 | Piscivorous birds | HV | -0.017 | 0.007 |
|  | 9 | Adult pollock 2+ | MF | 0.003 | 0.004 |
|  | 10 | Juvenile pollock 0-1 | MF | -0.014 | 0.031 |
|  | 11 | Other demersal fish | MF | -0.033 | -0.010 |
|  | 12 | Large Flatfish | MF | -0.093 | -0.075 |
|  | 13 | Small Flatfish | MF | 0.043 | -0.068 |
|  | 14 | Shallow Pelagics | MP | -0.014 | 0.004 |
|  | 15 | Deep Pelagics | MP | 0.047 | 0.031 |
|  | 16 | Deepwater fish | MF | -0.436 | 0.397 |
|  | 17 | Jellyfish | MF | -0.003 | 0.004 |
|  | 18 | Cephalopods | SS | -0.085 | 0.084 |
|  | 19 | Benthopelagic feeders | MF | -0.004 | 0.016 |
|  | 20 | Infauna | BI | -0.001 | -0.002 |
|  | 21 | Epifauna | BI | -0.011 | 0.013 |
|  | 22 | Large zooplankton | LZ | 0.003 | -0.007 |
|  | 23 | Herbivorous zooplankton | BI | -0.009 | 0.008 |
|  | 24 | Phytoplankton | NM | 0.000 | 0.000 |
|  | 25 | Discards | NM | 0.000 | 0.000 |
|  | 26 | Detritus | NM | 0.000 | 0.000 |
|  | F1 | Pollock trawl |  | 0.004 | 0.004 |
|  | F2 | cod trawl |  | -0.033 | -0.010 |
|  | F3 | Flatfish trawl |  | 0.016 | -0.070 |
|  | F4 | Herring roe |  | -0.014 | 0.004 |
|  | F5 | DW pelagic |  | -0.014 | 0.077 |
|  | F6 | Whaling |  | 0.477 | -0.476 |
|  | F7 | SeaLion cull |  | 0.487 | -0.478 |
| **Gulf of St. Lawrence** | 1 | Cetacea | HV | -0.045 | -0.649 |
|  | 2 | Harp seals | HV | -0.137 | -0.638 |
|  | 3 | Hooded seals | HV | -0.241 | -0.496 |
|  | 4 | Grey seals | HV | -0.139 | -0.696 |
|  | 5 | Harbour seals | HV | -0.159 | 0.105 |
|  | 6 | Seabirds | HV | -0.016 | -0.714 |
|  | 7 | Large cod | MF | -0.028 | -0.542 |
|  | 8 | Small cod | MF | -0.031 | 0.304 |
|  | 9 | Large Greenland halibut | MF | -0.115 | -0.486 |
|  | 10 | Small Greenland halibut | MF | -0.492 | 0.542 |
|  | 11 | American plaice | MF | -0.062 | 0.275 |
|  | 12 | Flounders | MF | 0.042 | 0.306 |
|  | 13 | Skates | MF | 0.217 | -0.027 |
|  | 14 | Redfish | MF | 0.035 | 0.110 |
|  | 15 | Large demersal fish | MF | -0.808 | 0.530 |
|  | 16 | Small demersal fish | MF | 0.007 | 0.059 |
|  | 17 | Capelin | SP | 0.010 | 0.106 |
|  | 18 | Sand lance | MF | 0.003 | -0.046 |
|  | 19 | Arctic cod | MF | -0.043 | -0.114 |
|  | 20 | Large pelagic fish | MP | -0.781 | 0.383 |
|  | 21 | Piscivorous small pelagic fish | SP | -0.188 | 0.141 |
|  | 22 | Planktivorous small pelagic fish | SP | -0.098 | -0.035 |
|  | 23 | Shrimp | BI | 0.087 | -0.147 |
|  | 24 | Large crustacea | BI | -0.014 | -0.146 |
|  | 25 | Echinoderms | BI | 0.101 | -0.228 |
|  | 26 | Molluscs | BI | 0.107 | -0.042 |
|  | 27 | Polychaetes | BI | 0.000 | 0.001 |
|  | 28 | Other bent. invert. | BI | 0.012 | -0.018 |
|  | 29 | Large zooplankton | LZ | -0.005 | -0.107 |
|  | 30 | Small zooplankton | BI | 0.002 | 0.026 |
|  | 31 | Phytoplankton | NM | 0.000 | 0.000 |
|  | 32 | Detritus | NM | 0.000 | 0.000 |
|  | F1 | Fisheries |  | -0.024 | -0.251 |
| **Benguela** | 1 | Phytoplankton | NM | -0.021 | 0.046 |
|  | 2 | Benthic producers | NM | -0.479 | -0.434 |
|  | 3 | Microzooplankton | BI | -0.050 | 0.045 |
|  | 4 | Mesozooplankton | BI | -0.008 | 0.006 |
|  | 5 | Macrozooplankton | LZ | 0.004 | -0.685 |
|  | 6 | Gelatinous zooplankton | BI | -0.231 | 0.083 |
|  | 7 | Anchovy | SP | -0.064 | -0.235 |
|  | 8 | Sardine | SP | -0.004 | 0.084 |
|  | 9 | Redeye | SP | -0.179 | 0.244 |
|  | 10 | Other small pelagic fish | SP | 0.132 | -0.176 |
|  | 11 | Chub mackerel | MP | -0.006 | -0.184 |
|  | 12 | Juvenile horse Mackerel | SP | 0.062 | 0.043 |
|  | 13 | Adult Horse mackerel | SP | 0.012 | -0.040 |
|  | 14 | Mesopelagic fish | MP | 0.003 | 0.020 |
|  | 15 | Snoek | MF | 0.029 | -0.071 |
|  | 16 | Other large pelagic fish | MP | -0.098 | -0.016 |
|  | 17 | Cephalopods | LS | -0.016 | 0.181 |
|  | 18 | Small *M. capensis* | MF | -0.085 | 0.063 |
|  | 19 | Large *M. capensis* | MF | -0.007 | -0.009 |
|  | 20 | Small *M. paradoxus* | MF | -0.009 | 0.018 |
|  | 21 | Large *M. paradoxus* | MF | 0.023 | -0.072 |
|  | 22 | Pelagic-feeding demersal fish | NM | -0.077 | 0.195 |
|  | 23 | Benthic-feeding demersal fish | NM | 0.003 | 0.003 |
|  | 24 | Pelagic-feeding chondrichthyans | NM | 0.006 | -0.059 |
|  | 25 | Benthic-feeding chondrichthyans | NM | 0.022 | -0.122 |
|  | 26 | Apex predatory chondrichthyans | NM | 0.038 | 0.044 |
|  | 27 | Seals | HV | 0.003 | -0.003 |
|  | 28 | Cetaceans | HV | 0.019 | -0.012 |
|  | 29 | Seabirds | HV | 0.001 | 0.005 |
|  | 30 | Meiobenthos | BI | -0.004 | 0.007 |
|  | 31 | Macrobenthos | BI | -0.002 | 0.003 |
|  | 32 | Detritus | NM | -0.001 | -0.003 |
|  | F1 | Purse seine |  | -0.012 | -0.163 |
|  | F2 | Midwater trawler |  | 0.000 | 0.103 |
|  | F3 | Demersal |  | 0.003 | -0.030 |
|  | F4 | Longlines |  | 0.004 | -0.651 |
|  | F5 | Crab traps |  | 0.006 | -0.059 |
|  | F6 | Lobster |  | 0.022 | -0.122 |
|  | F7 | Commercial linefishery |  | -0.207 | 0.035 |
|  | F8 | Recreational linefishery |  | -0.064 | -0.235 |
|  | F9 | Seal fishery |  | 0.521 | -0.434 |
|  | F10 | Other |  | -0.004 | 0.007 |
| **Eastern Tropical Pacific** | 1 | Pursuit Birds | HV | -0.017 | 0.620 |
|  | 2 | Grazing Birds | HV | 0.016 | 0.544 |
|  | 3 | Baleen Whales | HV | -0.008 | 0.008 |
|  | 4 | Toothed Whales | HV | -0.093 | -0.514 |
|  | 5 | Spotted Dolphin | HV | -0.023 | 0.486 |
|  | 6 | Meso Dolphin | HV | -0.004 | 0.579 |
|  | 7 | Sea Turtles | HV | -0.055 | 0.676 |
|  | 8 | Large yellowfin tuna | NM | 0.034 | -0.570 |
|  | 9 | Large bigeye tuna | NM | 0.068 | -0.602 |
|  | 10 | Large marlins | NM | -0.041 | -0.530 |
|  | 11 | Large sailfish | NM | -0.011 | -0.504 |
|  | 12 | Large swordfish | NM | 0.009 | -0.555 |
|  | 13 | Large dorado | MP | 0.110 | -0.417 |
|  | 14 | Large wahoo | NM | 0.095 | -0.629 |
|  | 15 | Large sharks | NM | 0.057 | -0.731 |
|  | 16 | Rays | MF | 0.081 | -0.728 |
|  | 17 | Skipjack tuna | MP | -0.337 | 0.083 |
|  | 18 | Albacore | MP | -0.538 | 0.253 |
|  | 19 | *Auxis* spp. | MF | 0.006 | 0.057 |
|  | 20 | Bluefin tuna | NM | -0.232 | 0.210 |
|  | 21 | Small yellowfin tuna | NM | -0.026 | -0.012 |
|  | 22 | Small bigeye tuna | NM | -0.730 | 0.318 |
|  | 23 | Small marlins | NM | -0.098 | 0.400 |
|  | 24 | Small sailfish | NM | 0.001 | 0.366 |
|  | 25 | Small swordfish | NM | 0.024 | 0.228 |
|  | 26 | Small dorado | MP | -0.240 | 0.095 |
|  | 27 | Small wahoo | NM | -0.797 | 0.505 |
|  | 28 | Small sharks | NM | -0.012 | -0.550 |
|  | 29 | Miscellaneous piscovores | MF | -0.120 | 0.213 |
|  | 30 | Flying fish | SP | 0.041 | -0.047 |
|  | 31 | Miscellaneous epipelagic fish | MF | 0.019 | -0.016 |
|  | 32 | Miscellaneous mesopelagic fish | MP | 0.015 | -0.025 |
|  | 33 | Cephalopods | SS | -0.046 | -0.003 |
|  | 34 | Crabs | BI | 0.016 | -0.013 |
|  | 35 | Mesozooplankton | LZ | -0.014 | 0.012 |
|  | 36 | Microzooplankton | BI | 0.009 | -0.007 |
|  | 37 | Large pytoplankton | NM | 0.000 | 0.000 |
|  | 38 | Small producers | NM | 0.000 | 0.000 |
|  | 39 | Detritus | NM | 0.000 | 0.000 |
|  | F1 | School Sets |  | -0.115 | -0.130 |
|  | F2 | Longliners |  | -0.052 | -0.441 |
|  | F3 | Floating Object Sets |  | -0.262 | -0.019 |
|  | F4 | Dolphin Sets |  | 0.016 | -0.458 |
|  | F5 | Bait Boats |  | -0.200 | 0.023 |
| **Gulf of Thailand** | 1 | *Rastrelliger* spp. | SP | -0.302 | -0.188 |
|  | 2 | *Scomberomorus* spp. | NM | -0.186 | 0.349 |
|  | 3 | Carangidae | MF | -0.466 | -0.002 |
|  | 4 | Pomfret | MF | -0.017 | 0.212 |
|  | 5 | Small pelagic fish | SP | -0.531 | -0.020 |
|  | 6 | False trevally | SP | -0.026 | 0.244 |
|  | 7 | Large piscivore fish | MF | -0.117 | -0.661 |
|  | 8 | Scianidae | MF | 0.078 | -0.734 |
|  | 9 | *Saurida* spp. | MF | -0.016 | -0.625 |
|  | 10 | Lutianidae | MF | -0.025 | -0.551 |
|  | 11 | Plectorhynchidae | MF | 0.055 | -0.194 |
|  | 12 | *Priacanthus* spp. | MF | 0.003 | -0.426 |
|  | 13 | *Sillago* spp. | MF | 0.001 | 0.066 |
|  | 14 | *Nemipterus* spp. | MF | 0.048 | -0.182 |
|  | 15 | Ariidae | MF | 0.041 | -0.166 |
|  | 16 | Rays | MF | 0.055 | -0.737 |
|  | 17 | Sharks | NM | -0.027 | -0.704 |
|  | 18 | Cephalopod | SS | -0.171 | -0.107 |
|  | 19 | Shrimps | BI | 0.056 | -0.155 |
|  | 20 | Crab, Lobster | BI | 0.001 | -0.013 |
|  | 21 | Trashfish | NM | -0.103 | -0.133 |
|  | 22 | Small demersal | MF | -0.147 | 0.155 |
|  | 23 | Demersal piscivore fish | MF | 0.004 | -0.074 |
|  | 24 | Demersal benthivore fish | MF | 0.020 | 0.282 |
|  | 25 | Shellfish | BI | 0.016 | -0.549 |
|  | 26 | Jellyfish | MF | 0.018 | 0.008 |
|  | 27 | Sea cucumber | BI | -0.055 | 0.737 |
|  | 28 | Seaweeds | NM | 0.000 | 0.000 |
|  | 29 | Coastal tuna | NM | 0.070 | -0.469 |
|  | 30 | Sergestid shrimp | BI | 0.136 | -0.024 |
|  | 31 | Mammals | HV | -0.255 | -0.043 |
|  | 32 | Pony fishes | MF | 0.157 | 0.244 |
|  | 33 | Benthos | BI | 0.000 | 0.003 |
|  | 34 | Zooplankton | LZ | 0.018 | 0.008 |
|  | 35 | Juvenile pelagic fish | SP | 0.189 | -0.174 |
|  | 36 | Juvenile *Caranx* spp. | MF | 0.131 | -0.109 |
|  | 37 | Juvenile *Saurida* spp. | MF | 0.092 | -0.387 |
|  | 38 | Juvenile *Nemipterus* spp. | MF | 0.092 | -0.444 |
|  | 39 | Phytoplankton | NM | 0.000 | 0.000 |
|  | 40 | Detritus | NM | 0.000 | 0.000 |
|  | F1 | Otter board trawl |  | -0.058 | -0.206 |
|  | F2 | Pair trawl |  | -0.042 | -0.129 |
|  | F3 | Beam trawl |  | -0.009 | -0.146 |
|  | F4 | Pushnet |  | -0.118 | -0.100 |
|  | F5 | Purse seine |  | -0.384 | -0.124 |
|  | F6 | Other gears |  | -0.018 | -0.402 |
| **North Sea** | 1 | Cod | MF | -0.105 | -0.392 |
|  | 2 | Haddock | MF | -0.001 | -0.424 |
|  | 3 | Herring | SP | 0.009 | -0.247 |
|  | 4 | Mackerel | SP | 0.000 | -0.552 |
|  | 5 | Norway pout | MF | 0.013 | -0.009 |
|  | 6 | Plaice | MF | -0.045 | -0.588 |
|  | 7 | Saithe | MF | -0.052 | -0.512 |
|  | 8 | Sandeel | MF | -0.001 | 0.251 |
|  | 9 | Sole | MF | 0.002 | -0.698 |
|  | 10 | Whiting | MF | -0.028 | -0.285 |
|  | 11 | Birds | HV | 0.003 | 0.161 |
|  | 12 | Gurnards | MF | 0.025 | -0.649 |
|  | 13 | Horsemackerel | MP | 0.005 | -0.856 |
|  | 14 | Other predator | MF | 0.006 | 0.068 |
|  | 15 | Raja | MF | 0.016 | -0.522 |
|  | 16 | Seals | HV | 0.006 | -0.698 |
|  | 17 | West mackerel | SP | 0.001 | -0.522 |
|  | 18 | Other invertebrates | BI | 0.004 | 0.043 |
|  | 19 | Juvenile cod | MF | 0.012 | 0.170 |
|  | 20 | Juvenile haddock | MF | 0.031 | 0.250 |
|  | 21 | Juvenile saithe | MF | 0.024 | -0.674 |
|  | 22 | Juvenile whiting | MF | 0.000 | 0.011 |
|  | 23 | Sprat | SP | -0.031 | -0.194 |
|  | 24 | Dab | MF | -0.054 | 0.362 |
|  | 25 | Copepods | BI | 0.001 | 0.010 |
|  | 26 | Euphausiids | LZ | -0.004 | -0.055 |
|  | 27 | Other crustaceans | BI | 0.004 | 0.076 |
|  | 28 | Echinoderms | BI | 0.006 | -0.101 |
|  | 29 | Polychaetes | BI | -0.001 | -0.057 |
|  | 30 | O.macrobenthos | BI | -0.005 | -0.042 |
|  | 31 | Phytoplankton | NM | 0.000 | 0.000 |
|  | 32 | Detritus | NM | 0.000 | 0.000 |
|  | F1 | Trawlers | F | -0.023 | -0.303 |
|  | F2 | Gill net | F | -0.041 | -0.401 |
|  | F3 | Seiners | F | 0.005 | -0.389 |
|  | F4 | Industrial | F | -0.001 | -0.024 |
| **Strait of Georgia** | 1 | Transient Orcas | HV | 0.289 | -0.214 |
|  | 2 | Dolphins (and Resident Orcas) | HV | -1.325 | 0.211 |
|  | 3 | Seals Sealions | HV | -0.367 | -0.469 |
|  | 4 | Halibut | MF | 0.082 | -0.581 |
|  | 5 | Lingcod | MF | -0.146 | -0.433 |
|  | 6 | Dogfish shark | MF | 0.126 | 0.402 |
|  | 7 | Adult hake | MF | -0.114 | 0.108 |
|  | 8 | Juvenile hake | MF | 0.000 | -0.102 |
|  | 9 | Adult resident Coho salmon | MF | -0.195 | -0.136 |
|  | 10 | Juvenile resident Coho salmon | MF | -0.307 | 0.382 |
|  | 11 | Adult resident Chinook salmon | MF | -0.152 | -0.265 |
|  | 12 | Juvenile resident Chinook salmon | MF | -0.202 | -0.204 |
|  | 13 | Demersal Fishes | MF | -0.067 | 0.056 |
|  | 14 | Sea Birds | HV | -0.024 | 0.005 |
|  | 15 | Small Pelagics | SP | 0.037 | 0.049 |
|  | 16 | Eulachon | SP | 0.029 | 0.077 |
|  | 17 | Adult herring | SP | 0.019 | -0.363 |
|  | 18 | Juvenile herring | SP | 0.044 | 0.208 |
|  | 19 | Jellyfish | MF | -0.001 | 0.008 |
|  | 20 | Predatory Invertebrates | BI | 0.040 | -0.015 |
|  | 21 | Shellfish | BI | -0.009 | -0.006 |
|  | 22 | Grazing Invertebrates | BI | 0.000 | -0.001 |
|  | 23 | Carnivorous zoolplankton | LZ | 0.021 | -0.020 |
|  | 24 | Herbivorous zoolplankton | BI | -0.007 | 0.021 |
|  | 25 | Kelp / Seagrass | NM | 0.000 | 0.000 |
|  | 26 | Phytoplankton | NM | 0.000 | 0.000 |
|  | 27 | Detritus | NM | 0.000 | 0.000 |
|  | F1 | Fisheries | F | 0.006 | -0.359 |
